# Supplementary material for: Genome-wide discovery of circulating cell-free DNA methylation signatures for the differential diagnosis of triple-negative breast cancer
Source: PeerJ. 2025 Aug 20;13:e19888. doi: 10.7717/peerj.19888 (PMC12374692; doi:10.7717/peerj.19888)
Supplement: Supplemental Information 1 [file peerj-13-19888-s001.docx]

**Supporting Information**

Fig. S1. Gene ontology enrichment analysis of differentially methylated genes between TNBC and non-TNBC tissues. 2

Fig. S2. The correlation among the 113 selected methylation markers. Blue indicates negative correlation, and red indicates positive correlation. 2

Fig. S3. Diagnostic effect of methylation diagnostic score (MDS) for different clinical stages of TNBC in TCGA dataset. 3

Fig. S4. Time-dependent ROC curves for evaluating the prognostic performance of cg06268921 in TNBC patients from the TCGA cohort. 4

Fig. S5. Forest plots of the meta-analysis for the association between cg06268921 methylation and overall survival (A) or disease-free survival (B) in the TCGA and GSE72251 TNBC cohorts. 4

Fig. S6. Optimization of annealing temperature for mddPCR assay. 5

Fig. S7. Analysis of limit of quantification (LOQ) for multiplex quantitative methylation-specific PCR (mqMSP assay) and mddPCR assay. 5

Fig. S8. Circulating cell free DNA concentrations in TNBC and non-TNBC plasma. 6

Fig. S9. The number of methylated copies of candidate genes in mddPCR assay. 7

Fig. S10. Diagnostic power of cfDNA-based MDS for different clinical stages TNBC in cfDNA validation cohort. 8

Fig. S11. Methylation levels of eight candidate markers in cfDNA from healthy individuals and luminal B breast cancer patients in GSE214344. 9

Table S1 Basic information of each breast cancer patient included in Infinium MethylationEPIC BeadChip analysis. 10

Table S2 Primer and probe sequences of candidate genes and reference gene. 11

Table S3 The clinical characteristics of patients in public datasets. 12

Table S4 Difference of eight CpG sites for distinguishing TNBC from non-TNBC tissues in TCGA dataset. 13

Table S5 Diagnostic performance of eight CpG sites for distinguishing TNBC from non-TNBC tissues in TCGA dataset. 13

Table S6 Sensitivity and specificity of the methylation diagnostic score in the TCGA and GSE69914 datasets. 13

Table S7 Univariate Cox analyses for overall survival of CpG sites in the TCGA-TNBC cohort. 14

Table S8 Univariate and multivariate Cox analyses for overall survival of cg06268921 in the TCGA and GSE72251 TNBC cohorts. 14

Table S9 Univariate Cox analyses for disease-free survival of CpG sites in the TCGA-TNBC cohort. 14

Table S10 Univariate and multivariate Cox analyses for disease-free survival of cg06268921 in the TCGA and GSE72251 TNBC cohorts. 15

Table S11 The mix preparation for one reaction. 15


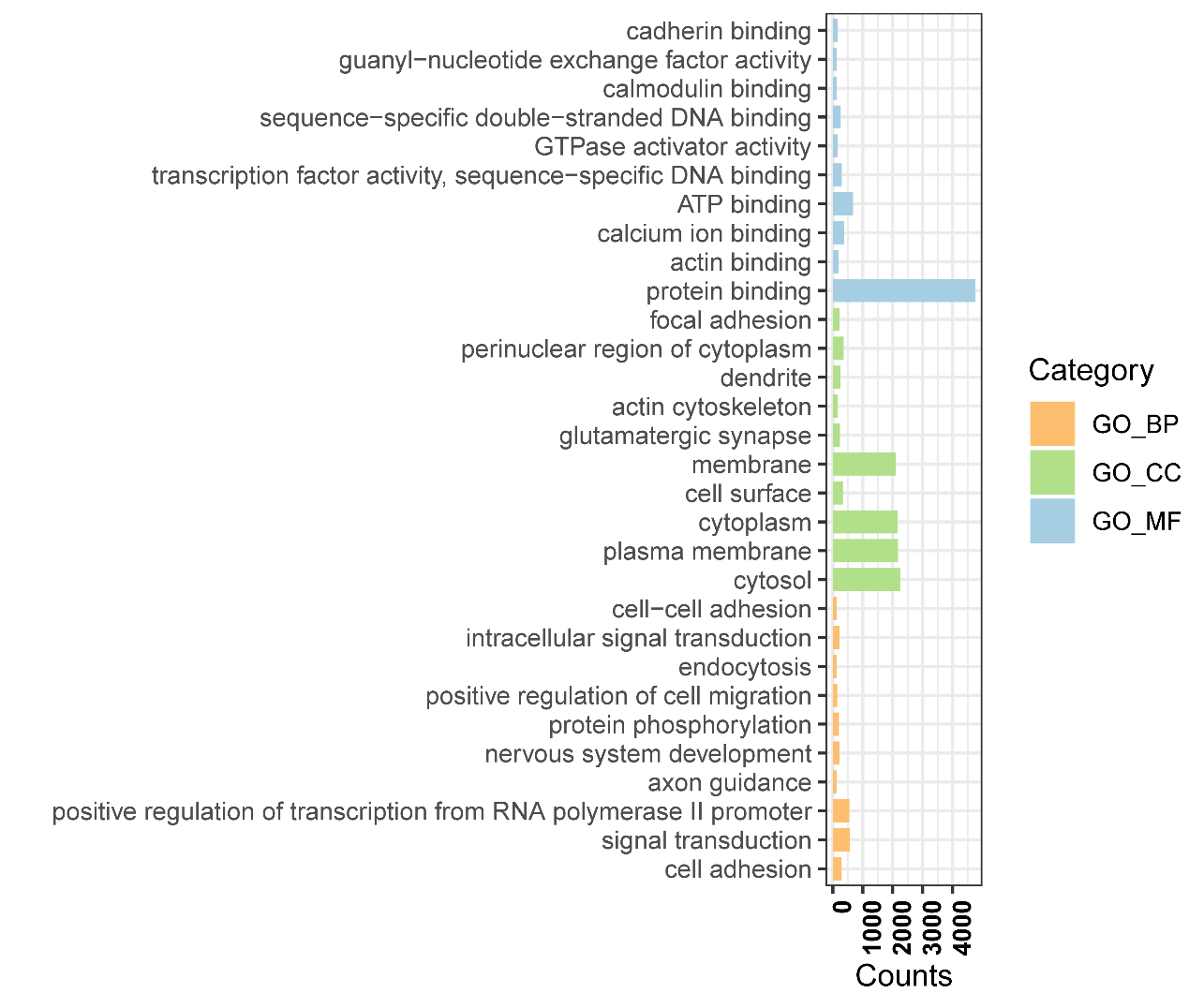


## Fig. S1. Gene ontology enrichment analysis of differentially methylated genes between TNBC and non-TNBC tissues.

**
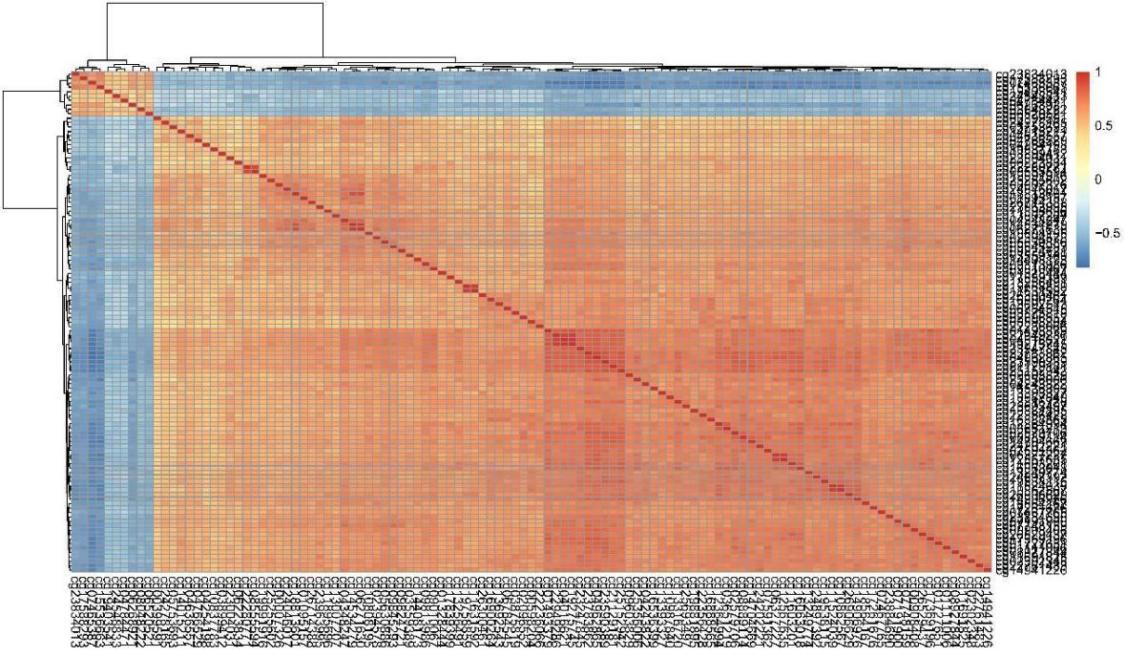
**

## Fig. S2. The correlation among the 113 selected methylation markers. Blue indicates negative correlation, and red indicates positive correlation.


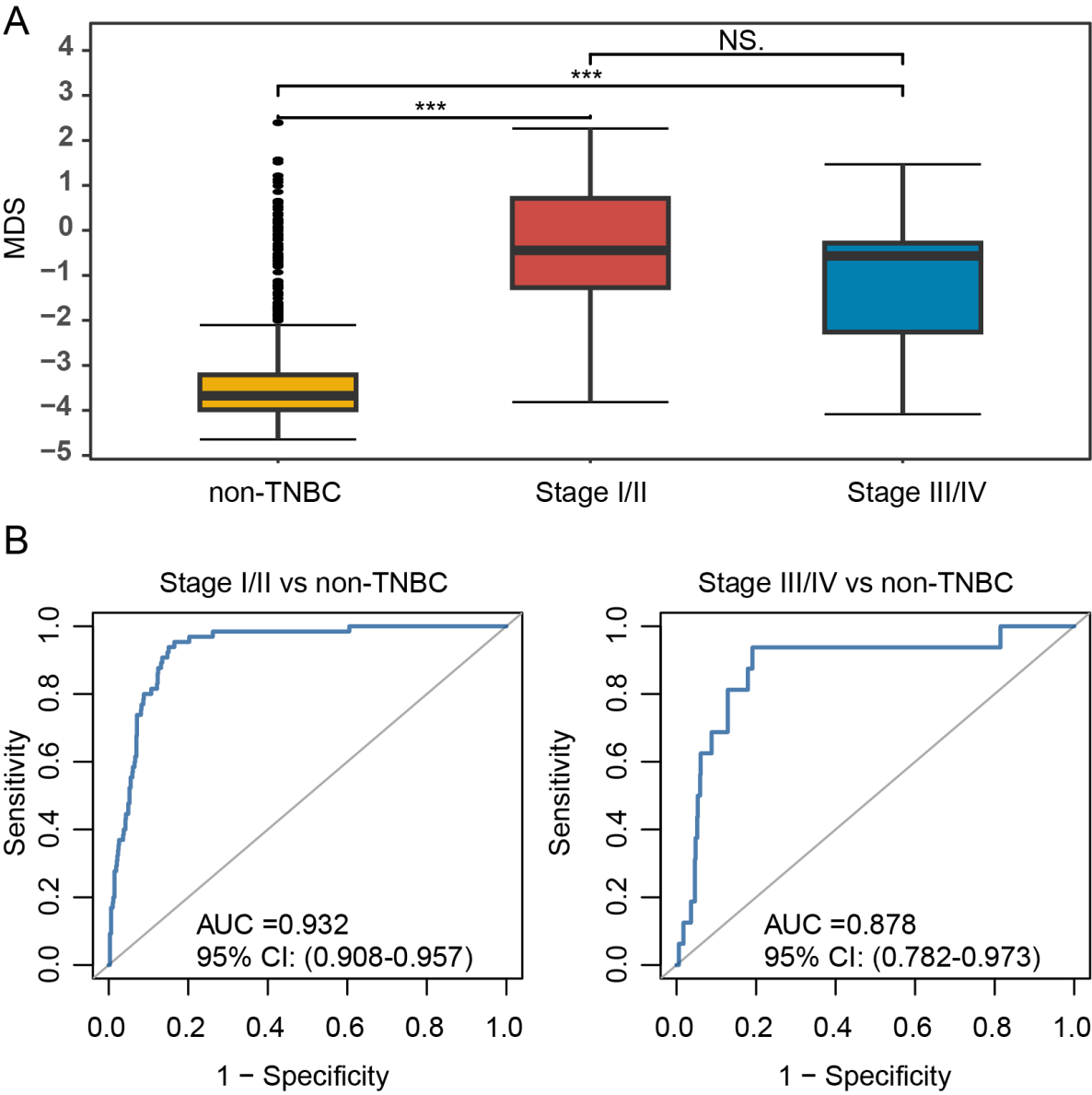


## Fig. S3. Diagnostic effect of methylation diagnostic score (MDS) for different clinical stages of TNBC in TCGA dataset.

A, Boxplot of MDS for non-TNBC and different stages of TNBC. B, Receiver operating characteristic curve of MDS for distinguishing TNBC with stage I/II and stage III/IV from non-TNBC, respectively.


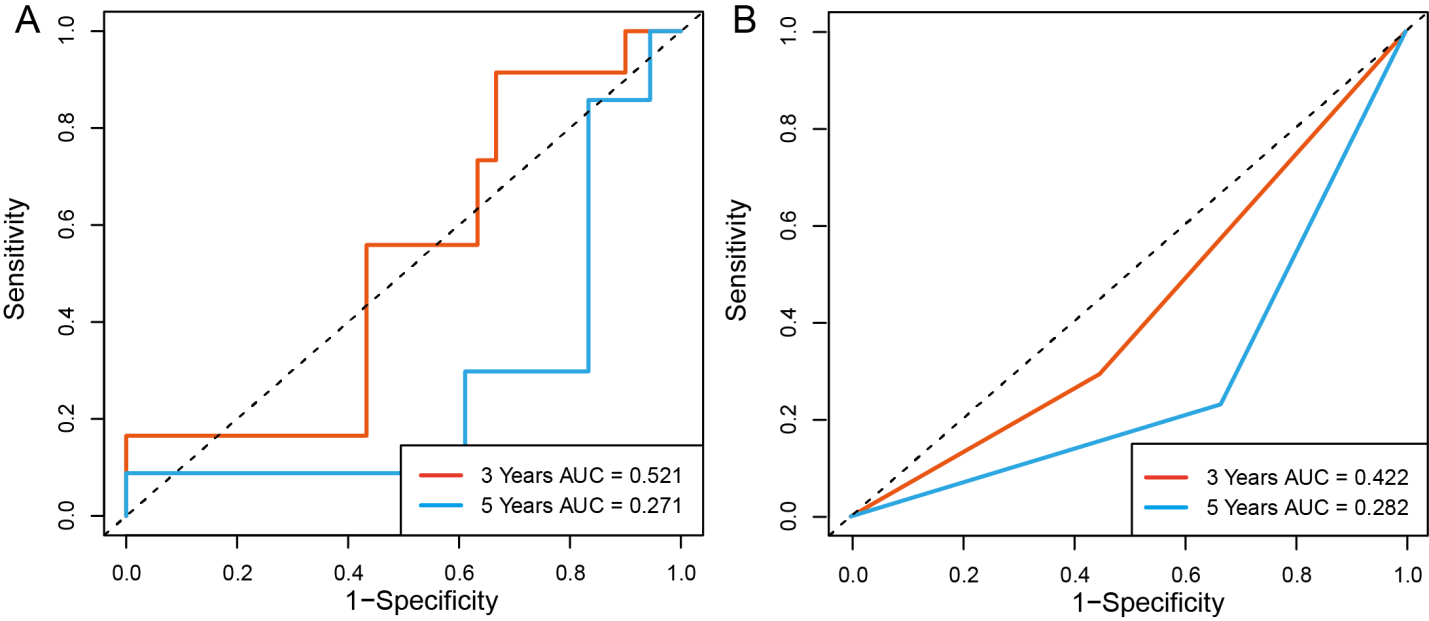


## Fig. S4. Time-dependent ROC curves for evaluating the prognostic performance of cg06268921 in TNBC patients from the TCGA cohort.

A, Time-dependent ROC curve for predicting 3-, 5-year overall survival; B, Time-dependent ROC curve for predicting 3-, 5-year disease-free survival. ROC, Receiver Operating Characteristic.


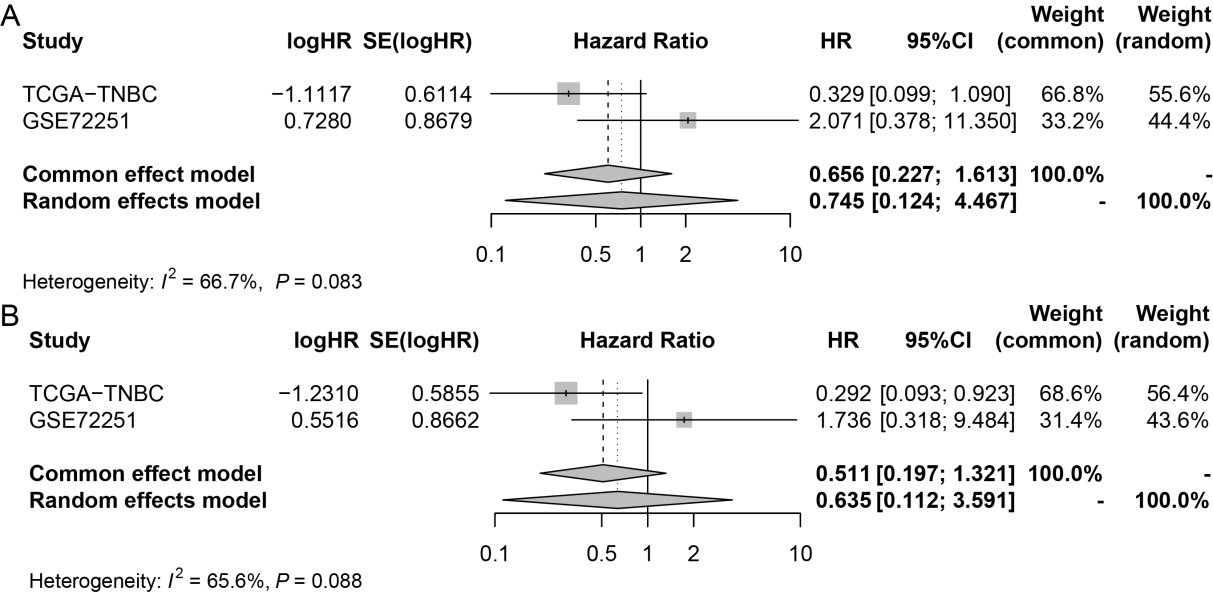


## Fig. S5. Forest plots of the meta-analysis for the association between cg06268921 methylation and overall survival (A) or disease-free survival (B) in the TCGA and GSE72251 TNBC cohorts.

Due to the lack of stage in GSE72251 cohort, hazard ratios were estimated using univariate Cox regression analysis.


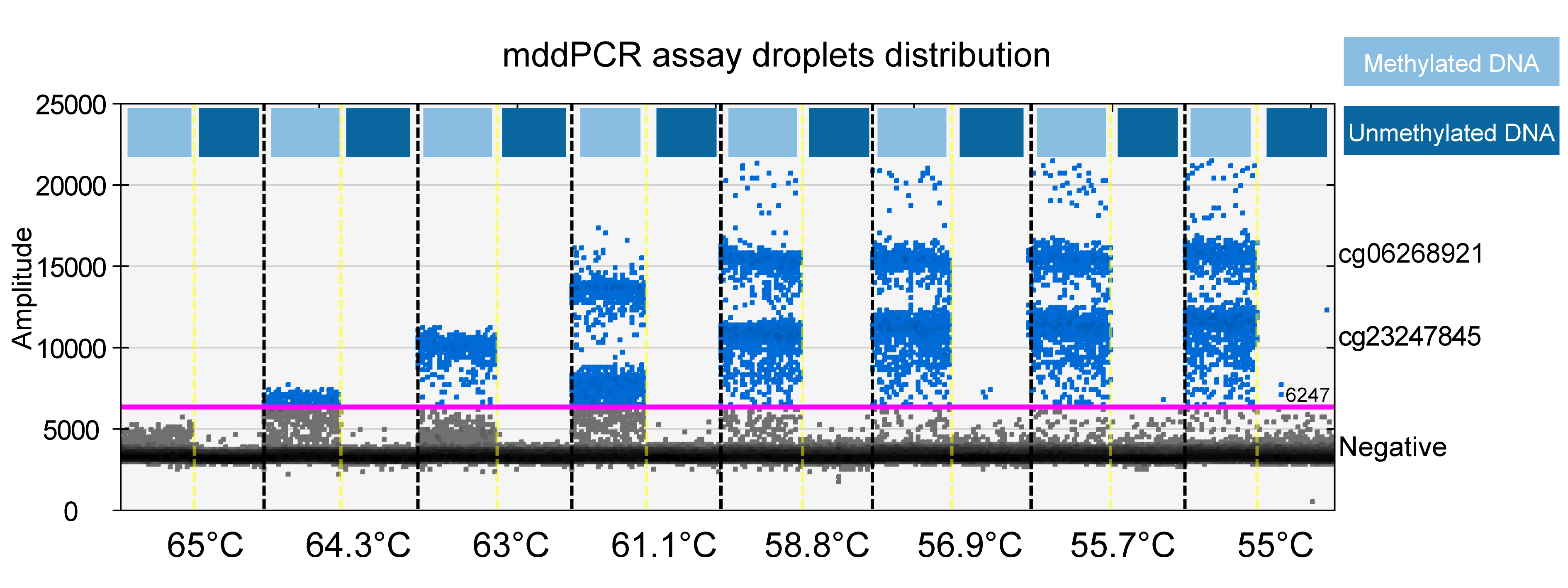


## Fig. S6. Optimization of annealing temperature for mddPCR assay.

For mddPCR assay, 58.8 °C was considered as the optimal annealing temperature. Each column (x-axis) indicating one PCR plate well, filling with methylated DNA and unmethylated DNA. Y-axis: fluorescence amplitude of droplet detection in the FAM channel.


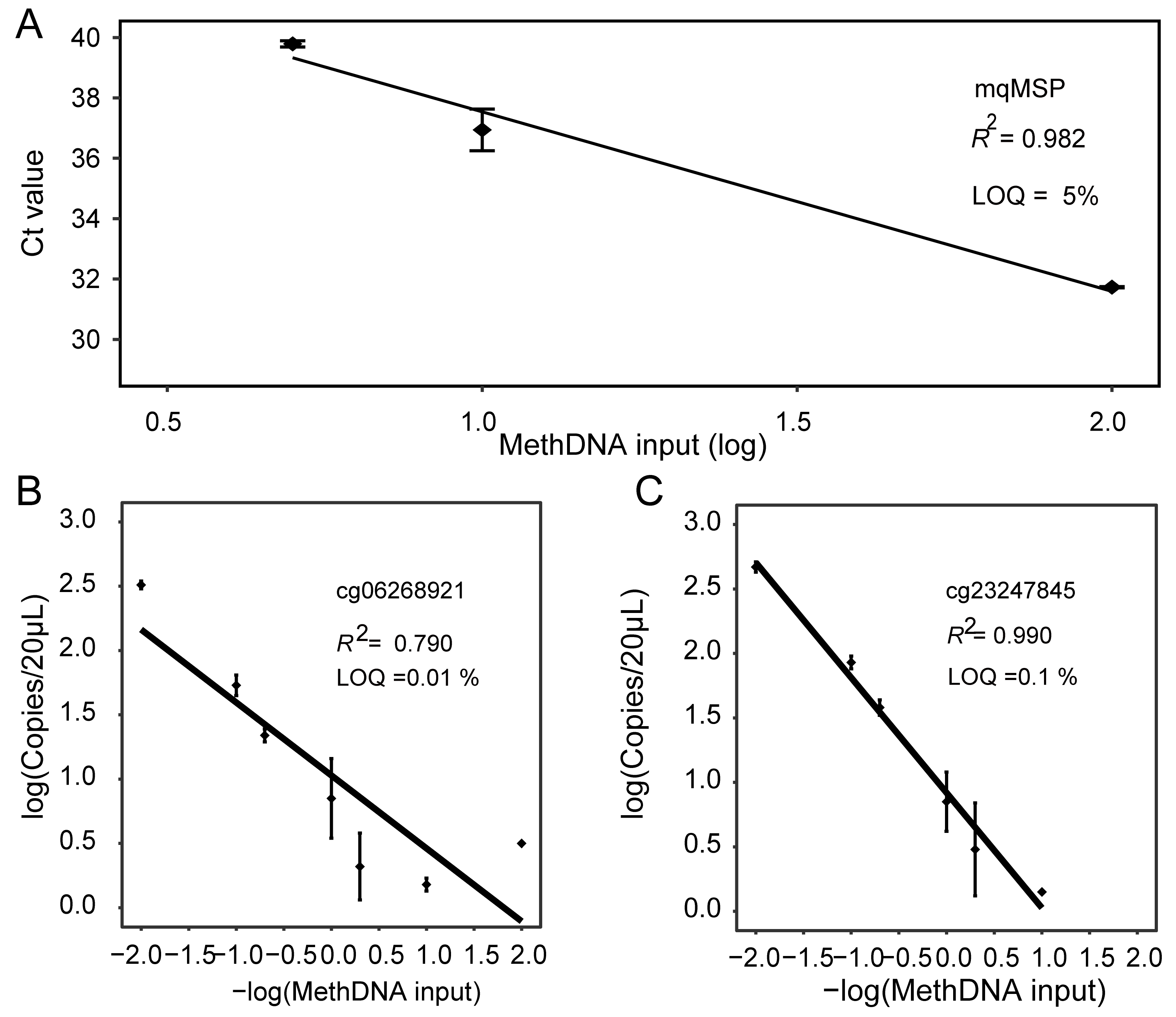


## Fig. S7. Analysis of limit of quantification (LOQ) for multiplex quantitative methylation-specific PCR (mqMSP assay) and mddPCR assay.

Calibration curves showing the linear dynamic range of mqMSP assay (A), cg06268921 (B), and cg23247845 (C) using the mixture of 10ng of the methylated DNA control with the unmethylated DNA control and diluting it at 100%, 10%, 5%, 1%, 0.5%, 0.1%, 0.01%, and 0.


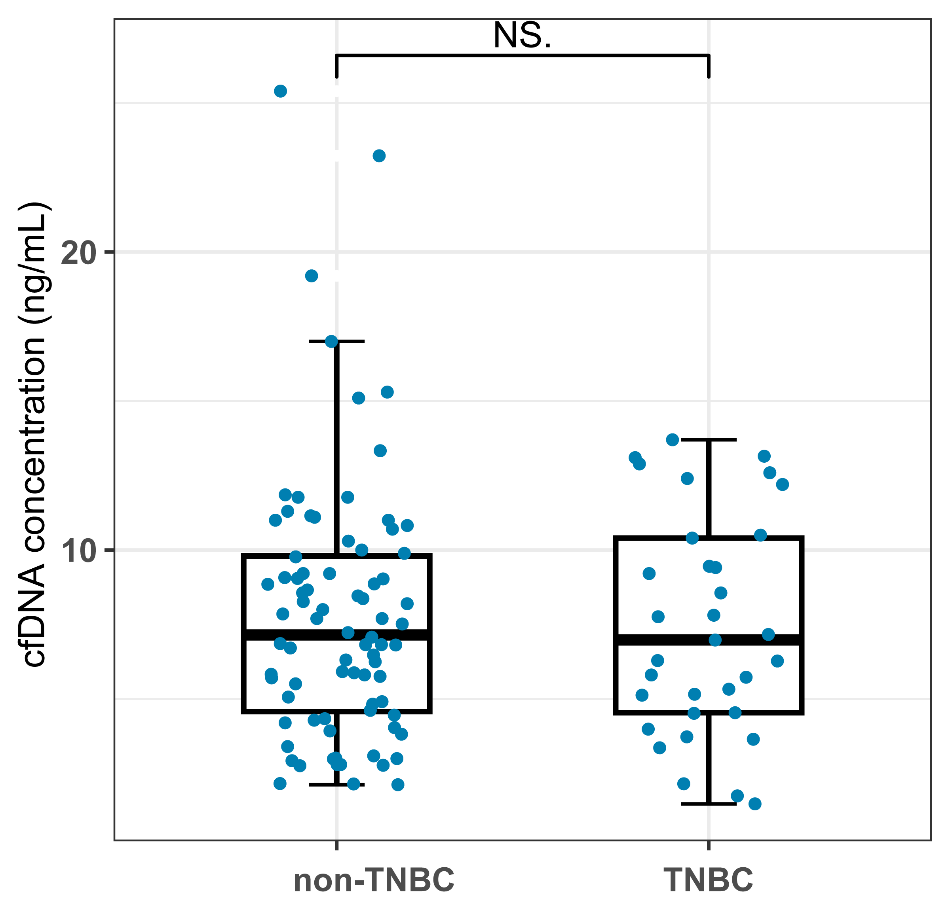


## Fig. S8. Circulating cell free DNA concentrations in TNBC and non-TNBC plasma.

NS, no significance.


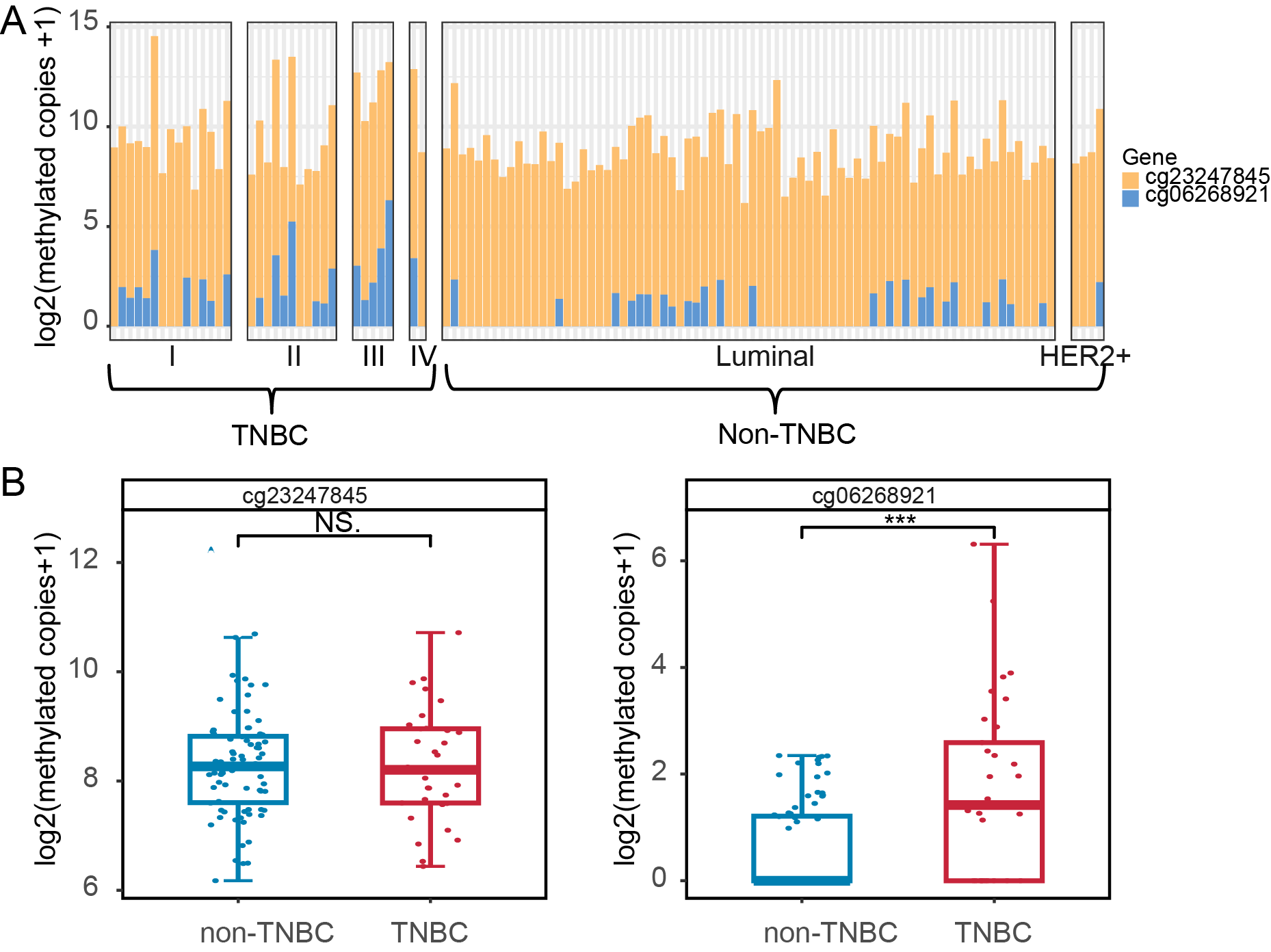


## Fig. S9. The number of methylated copies of candidate genes in mddPCR assay.

A, Barplot displaying data from the cfDNA samples. For each sample (X-axis), the height of the barplot indicating the number of methylated DNA copies per mL of plasma in the mddPCR assay, and the sum (Y-axis), and each colored segment representing an individual gene. B, Boxplot and dotplot of the number of methylated copies in mddPCR assay. ***: *P* ≤ 0.001. NS, no significance.


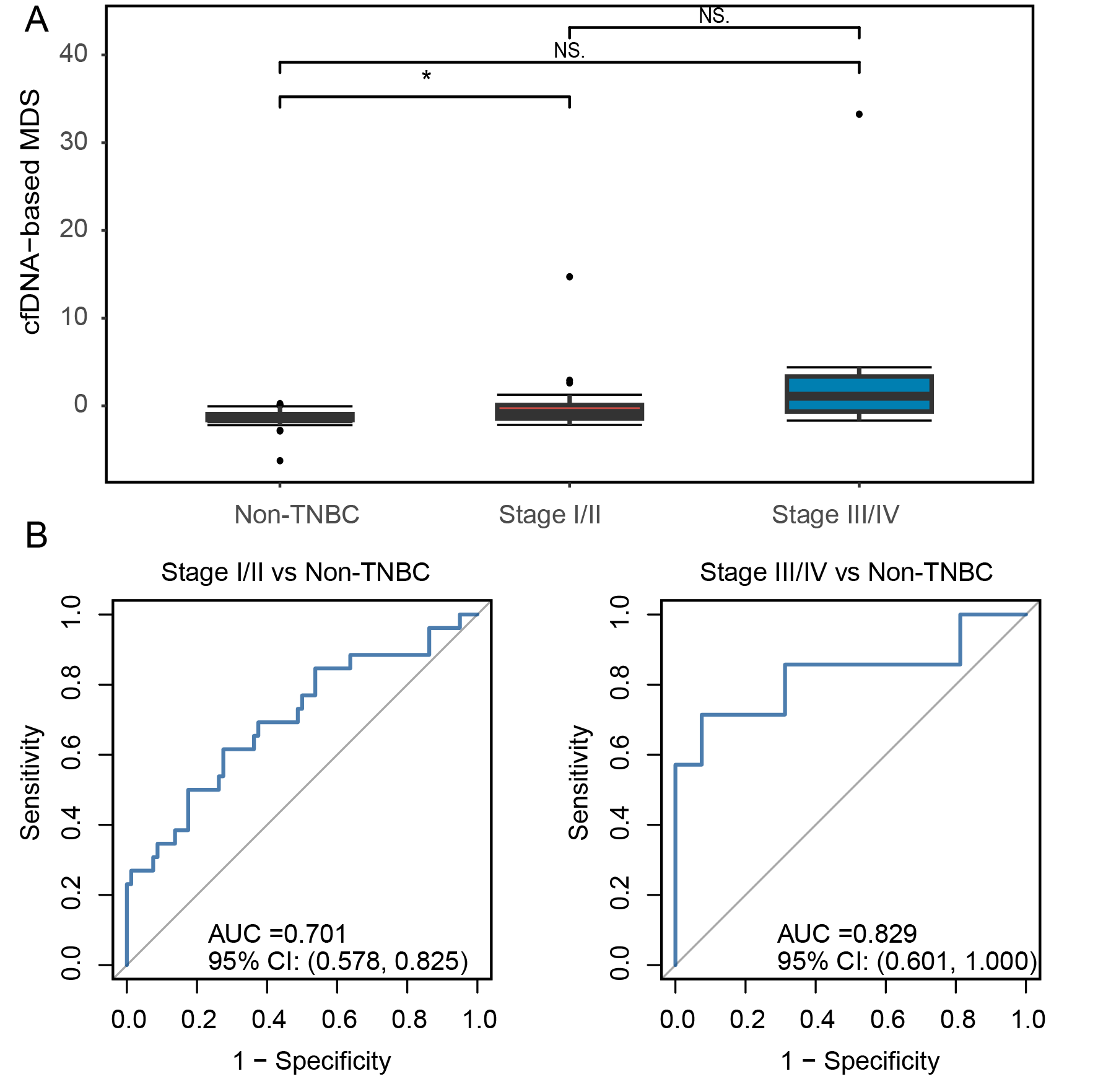


## Fig. S10. Diagnostic power of cfDNA-based MDS for different clinical stages TNBC in cfDNA validation cohort.

A, The cfDNA-based MDS in non-TNBC and different stages TNBC. B, Receiver operating characteristic curve of cfDNA-based MDS for distinguishing TNBC with stage I/II and stage III/IV from non-TNBC, respectively. *: *P* ≤ 0.05. NS, no significance.


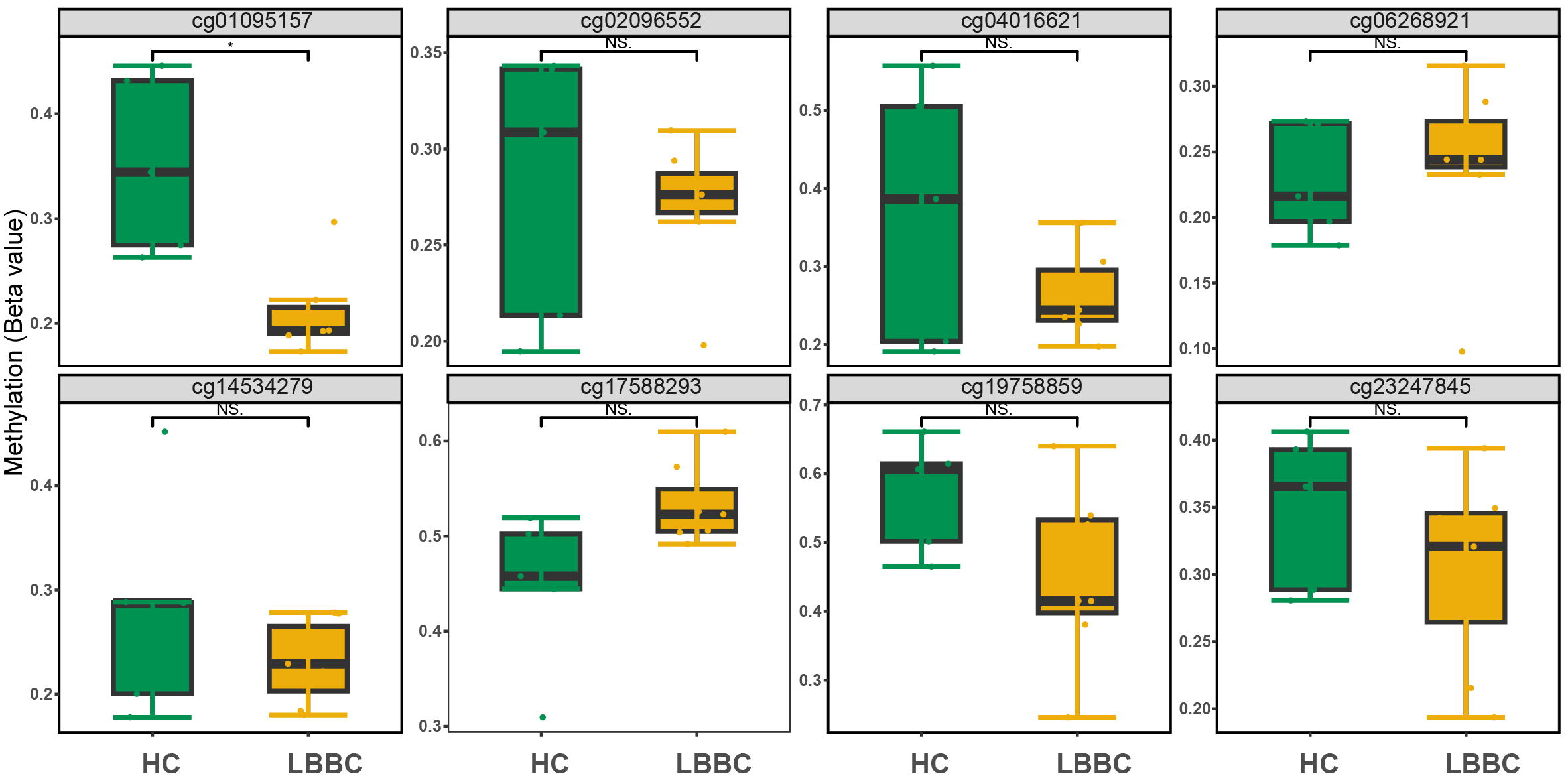


## Fig. S11. Methylation levels of eight candidate markers in cfDNA from healthy individuals and luminal B breast cancer patients in GSE214344.

HC, Healthy control; LBBC, luminal B breast cancer; *: *P* ≤ 0.05; NS, no significance.

## Table S1 Basic information of each breast cancer patient included in Infinium MethylationEPIC BeadChip analysis.

| Sample | Age | Height (cm) | Weight (kg) | Stage | Subtype | Xray | Ultrasound | Tumor_size | Lymph  node | T | N | M | ER | PR | Her-2 | FISH | ki67 |
| --- | --- | --- | --- | --- | --- | --- | --- | --- | --- | --- | --- | --- | --- | --- | --- | --- | --- |
| B003 | 58 | 160 | 53 | IV | TNBC | 5 | 5 | 4 | Positive | T2 | N2 | M1 | - | - | 0 | NA | 0.6 |
| B004 | 55 | 171 | 85 | II | non-TNBC | 5 | 4c | 2 | Positive | T1 | N1 | M0 | + | + | 3+ | NA | 0.6 |
| B007 | 55 | 165 | 70 | II | non-TNBC | 4b | 5 | 3.5 | Positive | T2 | N1 | M0 | + | - | 0 | NA | 0.3 |
| B008 | 59 | 158 | 70 | I | non-TNBC | 4a | 4b | 2 | Negative | T1 | N0 | M0 | + | + | 1+ | NA | 0.3 |
| B009 | 65 | 155 | 85 | IV | non-TNBC | 3 | 6 | 7 | Positive | T4 | N3 | M1 | + | + | 1+ | NA | 0.15 |
| B012 | 50 | 162 | 69 | I | non-TNBC | 4b | 4c | 2 | Negative | T1 | N0 | M0 | + | + | 0 | NA | 0.15 |
| B013 | 73 | 153 | 55 | IV | TNBC | 4c | 5 | 4 | Positive | T2 | N1 | M1 | - | - | 0 | NA | 0.6 |
| B014 | 34 | 175 | 70 | II | non-TNBC | 4a | 4c | 2.5 | Negative | T2 | N0 | M0 | + | + | 0 | NA | 0.4 |
| B015 | 58 | 165 | 74 | II | non-TNBC | 4a | 4c | 3 | Negative | T2 | N0 | M0 | + | + | 1+ | NA | 0.15 |
| B028 | 60 | 155 | 66 | II | non-TNBC | 4b | 4c | 3 | Negative | T2 | N0 | M0 | + | + | 2+ | positive | 0.4 |
| B034 | 62 | 160 | 65 | I | TNBC | 4b | 4b | 2 | Negative | T1 | N0 | M0 | - | - | 0 | NA | 0.8 |
| B036 | 69 | 156 | 60 | III | TNBC | NA | 4c | 6 | Positive | T3 | N2 | M0 | - | - | 0 | NA | 0.4 |
| B041 | 63 | 167 | 59 | II | TNBC | 4b | 4c | 3 | Positive | T2 | N1 | M0 | - | - | 0 | NA | 0.4 |
| B043 | 56 | 160 | 58 | I | non-TNBC | 4b | 4b | 1.7 | Negative | T1 | N0 | M0 | + | + | 3+ | NA | 0.5 |

## Table S2 Primer and probe sequences of candidate genes and reference gene.

| **Gene/Region** | **Primer/probe** | **Sequence (5'-3')** | **Tm ℃** | **GC%** | **Length (bp)** | **Product size (bp)** |
| --- | --- | --- | --- | --- | --- | --- |
| cg06268921 | Forward primer | TGGAGTTAATATTTAGG**CG**ATTTTTTG | 59 | 30 | 27 | 97 |
|  | MGB probe | TCGAACGACTCCCGC | 69 | 67 | 15 |  |
|  | Reverse primer | AACTTAAAACCCGAACCCCC | 57.9 | 50 | 20 |  |
| cg23247845 | Forward primer | TGTGGCGTATTTTTAGAGGGTTTT | 59 | 38 | 24 | 100 |
|  | MGB probe | ACGCTACCGAATTA**CG**CAT | 70 | 47 | 19 |  |
|  | Reverse primer | CGCGAATATATATATATCAATATAATCAAACG | 58.1 | 25 | 32 |  |
| ACTB | Forward primer | GGTTAAGTGTGATTTTGTGGTGTG | 57.2 | 42 | 24 | 80 |
|  | MGB probe | ACCCTCTACTACCC | 65 | 57 | 14 |  |
|  | Reverse primer | CCTTTTACAAAATTCACCCTCCT | 56.6 | 39 | 23 |  |

Note: These are the sequences after the bisulfite conversion; The CGs in bold were the sites detected in the Infnium HumanMethylationEPIC array; *ACTB* is the reference gene.

## Table S3 The clinical characteristics of patients in public datasets.

|  | **TCGA** | | **GSE69914** | | **GSE72251** |
| --- | --- | --- | --- | --- | --- |
| **Characteristic** | **TNBC (N = 83)** | **non-TNBC (N = 691)** | **TNBC (N = 30)** | **non-TNBC (N = 275)** | **TNBC (N = 34)** |
| Age, Mean (SD) | 55.2 (12.1) | 58.3 (13.3) | - | - | 55.4 (14.6) |
| ER, n (%) |  |  |  |  |  |
| Negative | 83 (100.0%) | 86 (12.4%) | 30 (100.0%) | 18 (6.5%) | 34 (100.0%) |
| Positive | 0 (0.0%) | 561 (81.2%) | 0 (0.0%) | 254 (92.4%) | - |
| Unknow | 0 (0.0%) | 44 (6.4%) | 0 (0.0%) | 3 (1.1%) | - |
| PR, n (%) |  |  |  |  |  |
| Negative | 83 (100.0%) | 154 (22.3%) | 30 (100.0%) | 51 (18.5%) | 34 (100.0%) |
| Positive | 0 (0.0%) | 490 (70.9%) | 0 (0.0%) | 217 (78.9%) | - |
| Unknow | 0 (0.0%) | 47 (6.8%) | 0 (0.0%) | 7 (2.5%) | - |
| HER2, n (%) |  |  |  |  |  |
| Negative | 83 (100.0%) | 309 (44.7%) | 30 (100.0%) | 194 (70.5%) | 34 (100.0%) |
| Positive | 0 (0.0%) | 90 (13.0%) | 0 (0.0%) | 43 (15.6%) | - |
| Unknow | 0 (0.0%) | 292 (42.3%) | 0 (0.0%) | 38 (13.8%) | - |
| Stage, n (%) |  |  |  |  |  |
| Stage I | 15 (18.1%) | 112 (16.2%) | - | - | - |
| Stage II | 50 (60.2%) | 381 (55.1%) | - | - | - |
| Stage III | 15 (18.1%) | 182 (26.3%) | - | - | - |
| Stage IV | 2 (2.4%) | 8 (1.2%) | - | - | - |
| Unknow | 1 (1.2%) | 8 (1.2%) | - | - | - |
| Vital status |  |  |  |  |  |
| Alive | 68 (81.9%) | 605 (87.6%) | - | - | 28 (82.4%) |
| Dead | 15 (18.1%) | 86 (12.4%) | - | - | 6 (17.6%) |

Note: All samples were from female participants.

## Table S4 Difference of eight CpG sites for distinguishing TNBC from non-TNBC tissues in TCGA dataset.

|  |  |  | **TCGA** |  |  | **GSE69914** |  |  |  |
| --- | --- | --- | --- | --- | --- | --- | --- | --- | --- |
| **CpG sites** | **Gene** | **TNBC** | **non-TNBC** | **Δβ** | ***P*** | **TNBC** | **non-TNBC** | **Δβ** | ***P*** |
| cg01095157 | *GORASP2* | 0.504 | 0.833 | -0.329 | 0.000 | 0.659 | 0.912 | -0.252 | 0.000 |
| cg02096552 | *DISP1* | 0.561 | 0.828 | -0.266 | 0.000 | 0.701 | 0.815 | -0.115 | 0.002 |
| cg04016621 | *GRK7* | 0.499 | 0.868 | -0.369 | 0.000 | 0.755 | 0.960 | -0.205 | 0.000 |
| cg06268921 | *NA* | 0.486 | 0.204 | 0.281 | 0.000 | 0.437 | 0.224 | 0.212 | 0.000 |
| cg14534279 | *NA* | 0.389 | 0.787 | -0.398 | 0.000 | 0.625 | 0.888 | -0.263 | 0.000 |
| cg17588293 | *ZBTB7B* | 0.622 | 0.343 | 0.279 | 0.000 | 0.534 | 0.352 | 0.182 | 0.000 |
| cg19758859 | *SASH1* | 0.533 | 0.834 | -0.301 | 0.000 | 0.617 | 0.794 | -0.177 | 0.000 |
| cg23247845 | *NA* | 0.481 | 0.841 | -0.360 | 0.000 | 0.734 | 0.915 | -0.181 | 0.000 |

Note: Δ*β* is the average *β* values of TNBC group minus the average *β* values of non-TNBC group.

## Table S5 Diagnostic performance of eight CpG sites for distinguishing TNBC from non-TNBC tissues in TCGA dataset.

| **CpG sites** | **Gene** | **Cut_off** | **AUC (95% CI)** | **Sensitivity** | **Specificity** |
| --- | --- | --- | --- | --- | --- |
| cg01095157 | *GORASP2* | 0.824 | 0.893 (0.867-0.919) | 95.2% | 74.7% |
| cg02096552 | *DISP1* | 0.742 | 0.824 (0.770-0.878) | 72.3% | 85.1% |
| cg04016621 | *GRK7* | 0.744 | 0.876 (0.834-0.919) | 80.7% | 88.4% |
| cg06268921 | *NA* | 0.273 | 0.809 (0.753-0.865) | 77.1% | 78.1% |
| cg14534279 | *NA* | 0.695 | 0.866 (0.826-0.906) | 90.4% | 78.6% |
| cg17588293 | *ZBTB7B* | 0.428 | 0.875 (0.836-0.914) | 92.8% | 76.7% |
| cg19758859 | *SASH1* | 0.751 | 0.829 (0.780-0.878) | 78.3% | 80.5% |
| cg23247845 | *NA* | 0.757 | 0.874 (0.829-0.919) | 86.7% | 85.5% |

Note: AUC, Area under the receiver operating characteristic curve; Sensitivity and specificity were determined based on cut_off values.

## Table S6 Sensitivity and specificity of the methylation diagnostic score in the TCGA and GSE69914 datasets.

|  | **TCGA** | | | | **GSE69914** | | | |
| --- | --- | --- | --- | --- | --- | --- | --- | --- |
|  | **Tested** | **Positive** | **Sensitivity** | **Specificity** | **Tested** | **Positive** | **Sensitivity** | **Specificity** |
| Stage I | 15 | 14 | 93.3% | - | - | - | - | - |
| Stage II | 50 | 47 | 94.0% | - | - | - | - | - |
| Stage III | 15 | 13 | 86.7% | - | - | - | - | - |
| Stage IV | 2 | 1 | 50.0% | - | - | - | - | - |
| Unknow | 1 | 1 | 100.0% | - | - | - | - | - |
| all-TNBC | 83 | 76 | 91.6% | - | 30 | 26 | 86.7% | - |
| non-TNBC | 691 | 587 | - | 84.0% | 275 | 248 | - | 90.2% |

Note: Sensitivity and specificity were determined based on -2.51 in two datasets.

## Table S7 Univariate Cox analyses for overall survival of CpG sites in the TCGA-TNBC cohort.

| **CpG sites** | **Risk c**ategories | HR | 95% CI | *P* |
| --- | --- | --- | --- | --- |
| cg19758859 | High (≥ 0.53) vs. Low (< 0.53) | 0.672 | 0.233-1.940 | 0.461 |
| cg01095157 | High (≥ 0.48) vs. Low (< 0.48) | 0.827 | 0.296-2.310 | 0.717 |
| cg14534279 | High (≥ 0.43) vs. Low (< 0.43) | 1.610 | 0.553-4.710 | 0.381 |
| cg17588293 | High (≥ 0.43) vs. Low (< 0.43) | 0.833 | 0.185-3.740 | 0.812 |
| cg06268921 | High (≥ 0.50) vs. Low (< 0.50) | 0.329 | 0.099-1.090 | **0.069** |
| cg04016621 | High (≥ 0.67) vs. Low (< 0.67) | 1.120 | 0.351-3.600 | 0.843 |
| cg23247845 | High (≥ 0.58) vs. Low (< 0.58) | 1.120 | 0.351-3.600 | 0.843 |
| cg02096552 | High (≥ 0.36) vs. Low (< 0.36) | 5.070 | 0.658-390 | 0.119 |

Note: *P <* 0.10 represents that CpG site is associated with overall survival.

## Table S8 Univariate and multivariate Cox analyses for overall survival of cg06268921 in the TCGA and GSE72251 TNBC cohorts.

|  | **Univariate analysis HR (95%CI)** | ***P*** | **Multivariate analysis HR (95%CI)** | ***P*** |
| --- | --- | --- | --- | --- |
| **TCGA-TNBC cohort** |  |  |  |  |
| Age |  |  |  |  |
| < 60/≥ 60 | 0.994 (0.957-1.032) | 0.752 | 0.998 (0.959-1.040) | 0.936 |
| Stage |  |  |  |  |
| I+II/III+IV | 2.76 (1.399-5.445) | 0.003 | 3.438 (1.553-7.611) | **0.002** |
| cg06268921 |  |  |  |  |
| ≥ 0.50 vs.< 0.50 | 0.329 (0.099-1.090) | 0.069 | 0.249 (0.064-0.966) | **0.044** |
| **GSE75067 cohort** |  |  |  |  |
| Age |  |  |  |  |
| < 60/≥ 60 | 1.275 (0.256-6.353) | 0.767 | 1.150 (0.224-5.907) | 0.867 |
| cg06268921 |  |  |  |  |
| < 0.50/≥ 0.50 | 2.071 (0.378-11.350) | 0.402 | 2.015 (0.358-11.358) | 0.427 |

Note: The analysis was adjusted for age and stage, GSE72251 cohort lacked the stage information.

## Table S9 Univariate Cox analyses for disease-free survival of CpG sites in the TCGA-TNBC cohort.

| **CpG sites** | **Risk categories** | **HR** | **95% CI** | ***P*** |
| --- | --- | --- | --- | --- |
| cg19758859 | High (≥ 0.53) vs. Low (< 0.53) | 0.667 | 0.248-1.790 | 0.422 |
| cg01095157 | High (≥ 0.48) vs. Low (< 0.48) | 0.548 | 0.201-1.490 | 0.239 |
| cg14534279 | High (≥ 0.43) vs. Low (< 0.43) | 1.160 | 0.420-3.230 | 0.771 |
| cg17588293 | High (≥ 0.43) vs. Low (< 0.43) | 0.621 | 0.176-2.200 | 0.460 |
| cg06268921 | High (≥ 0.50) vs. Low (< 0.50) | 0.292 | 0.093-0.923 | **0.036** |
| cg04016621 | High (≥ 0.67) vs. Low (< 0.67) | 1.160 | 0.401-3.340 | 0.788 |
| cg23247845 | High (≥ 0.58) vs. Low (< 0.58) | 1.190 | 0.411-3.420 | 0.752 |
| cg02096552 | High (≥ 0.36) vs. Low (< 0.36) | 2.360 | 0.537-10.400 | 0.255 |

Note: *P <* 0.10 represents that CpG site is associated with disease-free survival.

## Table S10 Univariate and multivariate Cox analyses for disease-free survival of cg06268921 in the TCGA and GSE72251 TNBC cohorts.

|  | **Univariate analysis HR (95%CI)** | ***P*** | **Multivariate analysis HR (95%CI)** | ***P*** |
| --- | --- | --- | --- | --- |
| **TCGA-TNBC cohort** |  |  |  |  |
| Age |  |  |  |  |
| < 60/≥ 60 | 0.908 (0.292-2.829) | 0.868 | 0.783 (0.245-2.502) | 0.680 |
| Stage |  |  |  |  |
| I+II/III+IV | 3.084 (1.640-5.798) | 0.003 | 4.197 (1.929-9.130) | **< 0.001** |
| cg06268921 |  |  |  |  |
| ≥ 0.50 vs.< 0.50 | 0.292 (0.093-0.923) | 0.036 | 0.194 (0.052-0.727) | **0.015** |
| **GSE75067 cohort** |  |  |  |  |
| Age |  |  |  |  |
| < 60/≥ 60 | 0.677 (0.124-3.698) | 0.652 | 0.666 (0.122-13.642) | 0.639 |
| cg06268921 |  |  |  |  |
| < 0.50/≥ 0.50 | 1.736 (0.318-9.484) | 0.524 | 1.760 (0.322-9.626) | 0.514 |

Note: The analysis was adjusted for age and stage, GSE72251 cohort lacked the stage information.

## Table S11 The mix preparation for one reaction.

|  | Multiplex ddPCR assay | |
| --- | --- | --- |
| Target region | cg06268921 | cg23247845 |
| Forward (20 μM) | 0.4 | 0.4 |
| Reverse (20 μM) | 0.4 | 0.4 |
| Taqman probe 6FAM (10 μM) | 0.9 | 0.75 |
| Taqman probe VIC (10 μM) |  | 0.75 |
| ACTB (reference gene) |  | |
| Forward (20 μM) | 0.4 | |
| Reverse (20 μM) | 0.4 | |
| Taqman probe VIC (10 μM) | 0.3 | |
| 2× ddPCR Supermix for Probes (no dUTP) | 10 | |
| DNA template (sample, µL) | 5-6 | |
| Volume input for droplet generation for ddPCR (µL) | 21 | |

Note: The working concentrations of primers and probes are 20 μM and 10 μM, and the probe solutions should be protected from light.
